# Characterization and Expression of Senescence Marker in Prolonged Passages of Rat Bone Marrow-Derived Mesenchymal Stem Cells

**DOI:** 10.1155/2016/8487264

**Published:** 2016-08-04

**Authors:** Noridzzaida Ridzuan, Akram Al Abbar, Wai Kien Yip, Maryam Maqbool, Rajesh Ramasamy

**Affiliations:** ^1^Stem Cell & Immunity Group, Immunology Laboratory, Department of Pathology, Faculty of Medicine and Health Sciences, Universiti Putra Malaysia, 43400 Serdang, Malaysia; ^2^Regenerative Medicine Research Program, Genetic and Regenerative Medicine Research Centre, Faculty of Medicine and Health Sciences, Universiti Putra Malaysia, 43400 Serdang, Malaysia; ^3^Clinical Genetics Unit, Department of Obstetrics & Gynecology, Faculty of Medicine and Health Sciences, Universiti Putra Malaysia, 43400 Serdang, Malaysia; ^4^Immunology Unit, Department of Pathology, Faculty of Medicine and Health Sciences, Universiti Putra Malaysia, 43400 Serdang, Malaysia

## Abstract

The present study is aimed at optimizing the in vitro culture protocol for generation of rat bone marrow- (BM-) derived mesenchymal stem cells (MSCs) and characterizing the culture-mediated cellular senescence. The initial phase of generation and characterization was conducted using the adherent cells from Sprague Dawley (SD) rat's BM via morphological analysis, growth kinetics, colony forming unit capacity, immunophenotyping, and mesodermal lineage differentiation. Mesenchymal stem cells were successfully generated and characterized as delineated by the expressions of CD90.1, CD44H, CD29, and CD71 and lack of CD11b/c and CD45 markers. Upon induction, rBM-MSCs differentiated into osteocytes and adipocytes and expressed osteocytes and adipocytes genes. However, a decline in cell growth was observed at passage 4 onwards and it was further deciphered through apoptosis, cell cycle, and senescence assays. Despite the enhanced cell viability at later passages (P4-5), the expression of senescence marker, *β*-galactosidase, was significantly increased at passage 5. Furthermore, the cell cycle analysis has confirmed the in vitro culture-mediated cellular senescence where cells were arrested at the G_0_/G_1_ phase of cell cycle. Although the currently optimized protocols had successfully yielded rBM-MSCs, the culture-mediated cellular senescence limits the growth of rBM-MSCs and its potential use in rat-based MSC research.

## 1. Introduction

Mesenchymal stem cells (MSCs) were originally isolated by Friedenstein and colleagues in guinea pig's bone marrow [1], characterized as multipotent adult stem cells that grow as foci with fibroblast-like morphology, termed as colony forming unit-fibroblasts (CFU-f). The early exploration and evaluation of MSC's biology were mainly achieved by exploiting bone marrow (BM) as a major source. To date, the common and efficient source of MSCs is remained to be the bone marrow. Although the frequency of MSCs within the bone marrow is very low (0.001–0.1% of mononuclear cells), MSCs are capable of extensive proliferation and expansion at in vitro culture [2].

It is now recognized that MSCs reside in many tissues and organs other than bone marrow [3] such as adipose tissue [4], liver [5], skeletal muscle [6], umbilical cord [7], umbilical cord blood [8], placenta [9], and synovium [10]. Besides human, MSCs have also been isolated from several animal species, namely, mouse [11–13], baboon [14], rat [15, 16], monkey [13], rabbit [17], dog [18], cat [19], and horse [20]. Despite the initial understanding on targeted/limited differentiation towards mesodermal lineages, the current studies have shown that, under a right stimulation, MSCs are able to differentiate into cells of endodermal and ectodermal origins such as *β*-pancreatic cells [21], hepatocyte [22], cardiomyocyte [23], skeletal muscle [24], neuron cells [25], and epithelial cells [26].

Many types of research that test the in vivo nature or functions of MSCs are still remarkably relying on animal models. Employing animals in biological science has become indispensable as most of the pharmacological and toxicological studies have been developed using laboratory-based animals as testing tools. For instance, the convenience of mice model with a possible genetic modification and availability of research reagents and species-specific antibodies ease the preclinical studies [27, 28]. Beside mouse model, some larger scale studies and disease-specific models were also developed using another type of animal such as rat, rabbit, fish, pig, and monkey [29–33]. In regard to current MSC research, human and mouse MSCs are extensively used in the present research scenario as these MSCs are relatively easy to be harvested and expanded at in vitro culture [34, 35]. When concerning other species, it has been reported that the rat MSCs are difficult to isolate and culture-expanded at the in vitro culture [15, 36, 37]. This conundrum drives the stem cells research using rat MSCs which is not as lucrative and well embraced as mouse counterpart. However, there is a need for the rat-based MSCs especially in diabetic research where most of the animal model investigations are tailored based on rats [38, 39]. One of the major concerns which arise in rat MSCs culture is to maintain a stable in vitro culture that provides an uninterrupted supply of stem cells for ongoing laboratory and animal works. Numerous research studies have reported the paucities of rBM-MSC culture where after certain rounds of passages the cells were failed to be amplified at adequate numbers. Despite this observation, the exact mechanism of such growth retardation is not fully elucidated. Thus, this research project is aimed at optimizing the laboratory protocol for isolation, characterization, and expansion of rat MSC to fill the gap in the current rat-based MSCs studies.

## 2. Materials and Methods

### 2.1. Animals

Sprague Dawley (SD) rats (250–350 g and 5–8 weeks) were obtained from Chenur Supplier (Kajang, Selangor, Malaysia). Animals were acclimatized and handled with standard animal care procedures as prescribed by Institutional Animal Care and Use Committee, Universiti Putra Malaysia (IACUC, UPM). Animals were sacrificed by cervical dislocation.

### 2.2. Generation of Rat Bone Marrow MSCs

Bone marrow cells were obtained from Sprague Dawley (SD) rats by flushing femurs and tibiae and cultured in 25 cm^2^ flask in a complete culture medium with 1% penicillin/streptomycin, 0.5% Fungizone, and 0.1% gentamycin (Gibco, United Kingdom). After 72 hours of plating, nonadherent cells were removed, and medium was replaced at every 48 hours. Adherent cells were further propagated and upon reaching 80–90% confluency, cells were trypsinised by using 0.05% trypsin-EDTA (Gibco, United Kingdom) at 37°C for 3–5 minutes. Harvested cells were cultured in 25 cm^2^ flasks for further expansion. During expansion period, media were changed every 2 days. Expanded cells were either used for downstream experiments or cryopreserved using freezing media (10% DMSO and 90% FBS). Media used in rBM-MSC expansion were LDMEM with GLUTAMAX-I (Gibco, United Kingdom), supplemented with 20% foetal bovine serum (FBS), 1% penicillin/streptomycin, 0.5% Fungizone, and 0.1% gentamycin (Gibco, United Kingdom). Supplements used in the optimization were 20 ng/mL basic fibroblast growth factor (bFGF) (R&D System USA), 1% nonessential amino acids (NEAA) (Gibco, United Kingdom), and 1% insulin transferrin sodium selenite (ITS) (Sigma Aldrich, USA).

### 2.3. Colony Forming Unit-Fibroblast Assay

Colony forming unit-fibroblast (CFU-f) assay was conducted with 1 × 10^6^ of nucleated cells from freshly isolated rat bone marrow and seeded in 60 mm^2^ cell culture dish (Becton Dickinson, USA) for 10 days. Various basal media were consumed to assess CFU-f as presented in Table 1 and purchased from Gibco, United Kingdom. Complete media were constituted with an individual basal medium supplemented with 1% penicillin/streptomycin, 0.5% Fungizone, and 0.1% gentamycin. Media change was conducted every 2 days of interval. The cells were stained with 1.5% crystal violet (Sigma Aldrich, USA) in 100% methanol and incubated for 10 minutes. The number of colonies more than 2 mm was considered and counted.

### 2.4. Immunophenotyping

The expression of cell surface markers was measured by a direct immunofluorescence staining and analysed by flow cytometer. Cells at passages 2-3 were trypsinised and cell count was performed using trypan blue exclusion test. Upon staining, cells were transferred into Fluorescence Activated Cell Sorting (FACS) tubes and washed with 1x phosphate buffer saline (1xPBS). Cells were labelled with fluorochrome conjugated mouse anti-rat antibodies (CD90.1-PE, CD45-PE, CD11B/C-PE, CD29-FITC, CD71-FITC, and CD44H-FITC) for 15 minutes at 4°C. For analysis, 10,000 cells were acquired by LSR Fortessa flow cytometer (BD Biosciences, USA) and analysed using FACS Diva Software (BD Biosciences, USA).

### 2.5. Differentiation Assays

 The adipocyte and osteogenic differentiation capabilities of passages 2-3 expanded rBM-MSCs were performed using StemPro adipogenesis differentiation kit (Gibco, Invitrogen, USA) and StemPro osteogenesis differentiation kit (Gibco, Invitrogen, USA), respectively, with minor modifications. Rat bone marrow mesenchymal stem cells were cultured in 60 mm^2^ Petri dish and incubated at 37°C in 5% CO_2_ humidified air. Upon reaching 100% confluency, cells were supplemented with respective differentiation medium where the inductive medium was changed every 2 days for 20 days. For adipogenic induction, cells were fixed in 4% paraformaldehyde and stained with Oil Red O solution whereas for osteogenic differentiation, cells were fixed in iced cold 70% ethanol and stained with Alizarin Red solution.

### 2.6. Reverse Transcription Polymerase Chain Reaction (RT-PCR)

Cellular differentiation of rBM-MSCs towards mesodermal lineages was further confirmed with gene expression assay using RT-PCR. Total RNA of cells that were cultured in either normal medium or induction media (adipo and osteo) retrieved using TRIzol reagent kit (Invitrogen, USA). DNase treatment was carried out by adding 20.5 *μ*L RNA with 2.5 *μ*L NE buffered DNase 1, 1 *μ*L of RNase inhibitor, and 1 *μ*L DNase 1 and incubated for 30 minutes at 37°C. Subsequently, 0.5 *μ*L of 0.25 M ethylene diamine tetraacetic acid (EDTA) was added and incubated for 10 minutes at 75°C. The RNA concentration was determined by using spectrophotometer. The cDNA was obtained by reverse transcription of 4 *μ*g of total RNA using 1 *μ*L oligo dT primer. Primers for PCR were adopted from the literature [16, 18, 40–42] as shown in Table 2. The detailed PCR procedures for cDNA were followed per Table 3.

### 2.7. Tritiated Thymidine Incorporation Assay

The proliferation of rBM-MSCs was determined by tritiated thymidine (3H-TdR) assay (Perkin Elmer, USA). The radioactive nucleotide 3H-TdR integrates only into actively proliferating cells during DNA synthesis, and the amount of 3H-TdR measured is directly proportional to the cell proliferation. Five thousand cells per well were cultured in 96-well plate for 24 h, 48 h, and 72 h. Cultures were pulsed with 10 *μ*L 3H-TdR (0.5 *μ*Ci/well) at 24 h prior to measurement. At the end of each time point (24 h, 48 h, and 72 h) cells were harvested onto glass filter mat (Perkin Elmer, USA) by using a 96-well plate automated cell harvester (Harvester Mach III M, TOMTEC, USA). The filter mat was dried using oven (Penasonic, Malaysia) (120°C) for 10 min before adding 5 mL scintillation fluid (OptiPhase Supermix Cocktail; Perkin Elmer, USA). The filter mat was then sealed and fitted into a scintillation cassette for radioactive measurement using luminescent Microbeta counter (MicroBetaTrilux). Results were expressed as count per minute (CPM).

### 2.8. Growth Kinetic and Doubling Time

Growth kinetic of rBM-MSCs was performed using trypan blue exclusion test. Approximately, 150,000 cells were plated into 6-well plate. Cells were grown for 6 days with medium change which was performed at every 3 days. Cells were harvested every day and counted. Growth kinetic curves of rBM-MSCs from different passages were plotted. The initial seeding number, days in culture, and yield of MSC cultures were recorded and computed into doubling time. Doubling time was determined by Patterson Formula 1 and expressed as mean doubling time:

(∗) $$\begin{array}{c} T_{d}=\frac{T\log2}{\log\left(N_{t}/N_{0}\right)}, \end{array}$$

where *T*_*d*_ is the doubling time (h), *T* is the time during which cells proliferated from *N*_0_ to *N*_*t*_ (h), and *N* is the cell count.

### 2.9. Apoptosis Assay

Apoptosis assay was performed using Annexin V/Dead Cell Apoptosis kit with FITC conjugated Annexin V and PI (Invitrogen, USA). Annexin V is Ca^2+^-dependent phospholipid binding protein that binds to phospholipid such as phosphatidylserine (PS). Annexin V along with propidium iodide (PI) allows identification of early apoptotic cells (PI negative; FITC Annexin V positive). Viable cells with intact membranes exclude PI, whereas membranes of dead and damage cells are permeable to PI [43]. Approximately 100,000 cells were washed with 1x Annexin binding buffer (ABB) and stained with 2 *μ*L Annexin V and 1 *μ*L PI for 15 min at room temperature. Cells were resuspended in 500 *μ*L 1xABB and acquired using LSR Fortessa flow cytometer (BD Bioscience, USA). For analysis, 10,000 cells were acquired and analysed using FACS Diva Software.

### 2.10. Senescence Associated *β*-Galactosidase (SA *β*-gal) Staining

Acid *β*-D-galactosidase is a hydrolase localized in lysosome which cleaves *β*-linked terminal galactosyl residues from substrates such as gangliosides, glycoproteins, and glycosaminoglycans [43]. Mammalian cells expressed lysosomal*β*-galactosidase (*β*-gal) activity at pH 4.0. Meanwhile, SA *β*-gal activity can be detected at pH 6 in senescence cells but not in proliferating cells. Both lysosomal *β*-gal and SA *β*-gal can be detected in situ by a cytochemical staining using chromogenic substrate 5-bromo-4-chloro-3-indolyl *β*-D-galactopyranoside (X-gal) [44]. X-gal cleaved by *β*-gal to produce insoluble blue precipitates [45]. Cells at passage two and passage five were fixed with 70% ethanol for 10 minutes and stained with X-gal in its respective buffer (pH 4 and pH 6) overnight before viewing under the phase contrast microscope. Senescent cells were evidenced by the formation of blue precipitate within the cells.

### 2.11. Cell Cycle Analysis

Cell cycle analysis of rBM-MSC was determined by measuring DNA content using PI dye. Cells at passage 2 and passage 5 were cultured in 25 cm^2^ flasks. Upon reaching 80–90% confluency, cells were harvested and fixed with 70% ethanol and subjected to overnight incubation at −20°C. Fixed cells were washed with 1xPBS and incubated with 0.5 mL staining buffer which consisted of 100 *μ*g/mL PI (Molecular Probe, Invitrogen) and 20 ng/mL RNase (Sigma) in 1xPBS for 30 minutes. At least 10,000 cells were acquired by flow cytometer and analysed via ModFit LT software (Verity Software House, USA).

### 2.12. Statistical Analysis

Values for all measurements were presented as mean ± SD unless otherwise stated. Comparison was performed by Student's* t*-test and one-way ANOVA. Significance levels were set at value *p* ≤ 0.05.

## 3. Results

### 3.1. Optimization of rBM-MSC Culture

**Figure 1:**
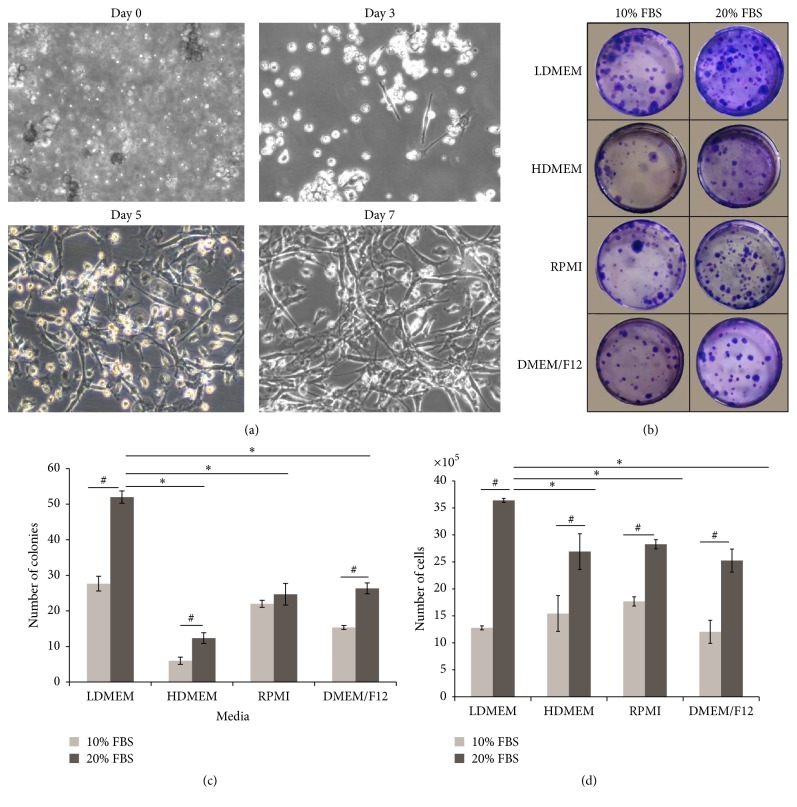
Generation and optimization of rBM-MSCs culture. Bone marrow was harvested from femur and tibia of SD rats and nucleated cells were cultured in T25 flask in day 0. By day 3, cells began to attach and heterogeneous population with predominant fibroblast-like morphology were observed by day 7 (a). One million of nucleated cells from bone marrow were cultured for 10 days in respective media and FBS concentrations. Colonies were subjected to crystal violet staining and colonies which brightly stained were counted (b). Four different basal media with 10% and 20% FBS concentration were utilized to culture 1 × 10^6^ freshly isolated BM nucleated cells for CFU and proliferation assays. CFU-f and proliferation assays were measured using crystal violet staining and trypan blue exclusion test, respectively. Results were representative of three independent experiments. ^*∗*^Significant values were compared between LDMEM 20% and other basal media with 20% FBS; ^#^significant values were compared between 10% FBS and 20% FBS of the respective media. *p* ≤ 0.05. Microscopic magnification: 200x.

Upon in vitro culture, single cells of rat BM have started to form adherent cell colonies from day 3 onwards. The colony of spindle-shaped cells has profoundly increased in size at day 5 and day 7 (Figure 1(a)). To determine the optimal media for the growth of rBM-MSCs, several basal media and two concentrations of FBS were tested for the ability to support the growth of colony forming unit-fibroblast and cell expansion.  Figure 1(b) shows the stained CFU-f of LDMEM, HDMEM, RPMI, and DMEM/F12 basal media supplemented with 10% FBS or 20% FBS, respectively. Regardless of the types of basal media, 20% supplemented FBS yields the highest number of colonies as compared to 10% FBS. Among all basal media, LDMEM reaps the highest number of colonies (CFU-f = 52), followed by DMEM/F12 (CFU-f = 26), RPMI (CFU-f = 24), and HDMEM (CFU-f = 12) (Figure 1(c)). To verify whether the number of colonies formed is accompanied by the total cell numbers, BM cells from passage 0 were cultured in respective basal media and serum concentrations. The number of expanding cells was calculated using trypan blue exclusion test at stipulated time points. As evidenced in CFU-f assay, the total cell counts are greater when 20% of FBS was consumed, whereas in terms of the type of basal medium, LDMEM induced a higher cell proliferation as compared to HDMEM, RPMI, and DMEM/F12 (Figure 1(d)).

### 3.2. Characterization of rBM-MSC

**Figure 2:**
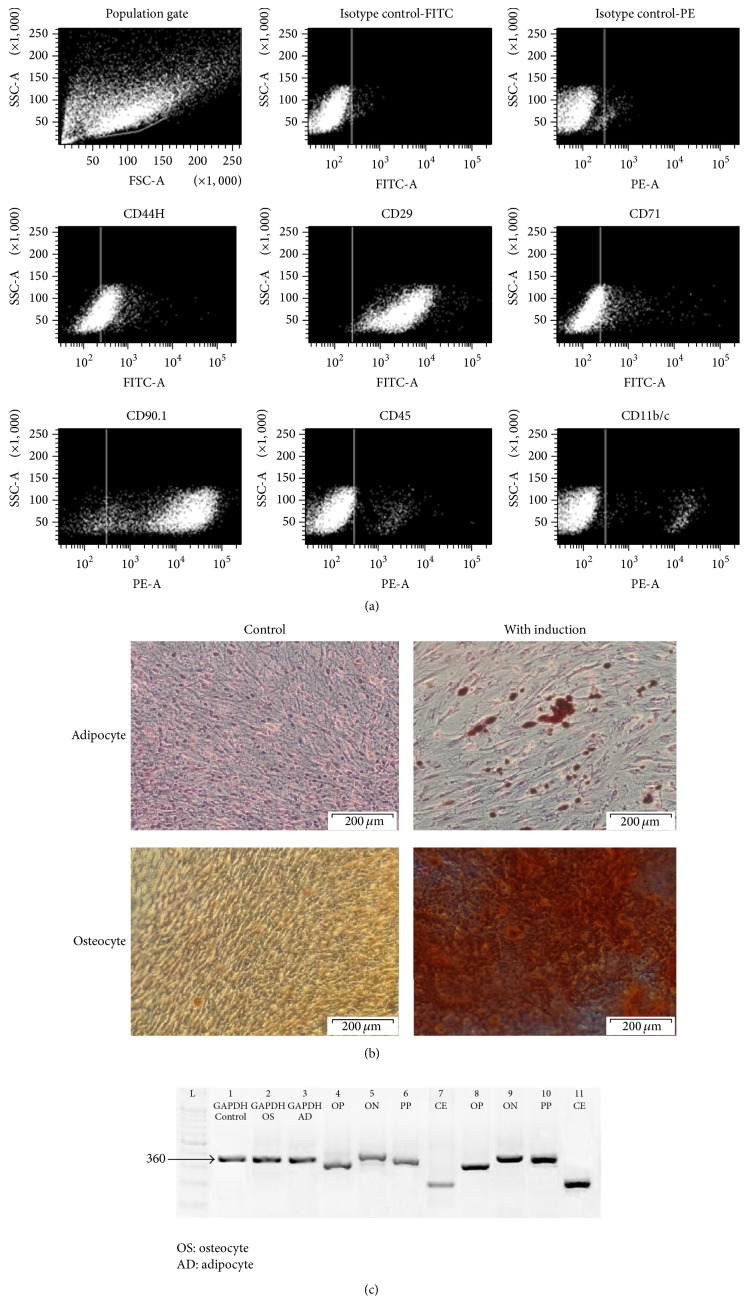
Characterization of rBM-MSCs. The immunophenotyping to characterize the surface markers was performed using rBM-MSC from passage 3. Cells were positively expressed as CD90.1, CD29, CD71, and CD44 and negatively expressed as CD11b/c and CD45 (a). Mesodermal differentiation was conducted using cells of passage 3 that is subjected to the relevant induction media. After 20 days of induction, cells were stained with Oil Red O solution and Alizarin Red solution, respectively. Adipogenic differentiation was evidence by lipid droplet formation stained with Oil Red O whereas osteogenic differentiation was evidenced by calcium deposits stained with Alizarin Red (b). Gene expression of differentiated adipocytes and osteocytes was evaluated using RT-PCR. Untreated rBM-MSC (control) showed faint expression for osteocytes (c). Results were representative of 3 experiments. OP: osteopontin, ON: osteonectin, PP: PPAR*γ*, and CE: C/EBPA. Microscopic magnification: 200x.

To analyse the expression of cell surface markers on rBM-MSCs, cells at passage 3 were subjected to the immunophenotyping. Flow cytometry result showed that rBM-MSCs are unequivocally positive for CD90.1 (94.8%), CD44H (41.6%), CD29 (99.7%), and CD71 (12.7%) and negative for hematopoietic markers CD45 (4.0%) and CD11b/c (4.3%) as shown in Figure 2(a). To assess the mesodermal differentiation ability of rBM-MSCs, cells at passage 3 were grown to the confluency and induced to differentiate into adipocytes and osteocytes using relevant induction media. Following 20 days of adipogenic induction, lipid vacuoles were detected by positive staining of Oil Red O whereas osteogenic differentiation was detected by positive staining of Alizarin Red solution (Figure 2(b)). Cell cultured in expansion media (negative control) showed neither detectable lipid vacuoles nor calcium deposition. To further confirm the mesodermal differentiation, gene expression analysis of adipocytes and osteocytes specific genes was conducted using RT-PCR. The selected adipocytes (PPAR*γ* and CEBP/A) and osteocytes gene (osteopontin and osteonectin) were analysed. Undifferentiated rBM-MSC (negative control) showed a faint expression of adipocytes and osteocytes genes. Differentiated rBM-MSCs (osteocytes and adipocytes) showed higher expression of adipocytes and osteocytes genes as compared to control as shown in Figure 2(c).

### 3.3. Growth Kinetics and Doubling Time of rBM-MSC

**Figure 3:**
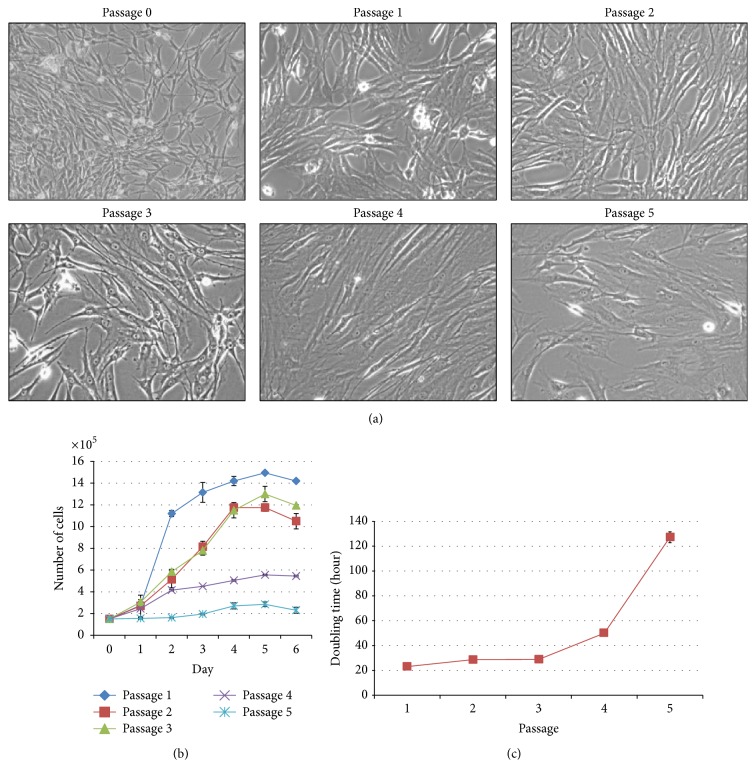
Growth kinetics and doubling time of rBM-MSC. Morphological observation of rBM-MSC cultured in LDMEM (20% FBS) in the presence of 20 ng/mL, 1% ITS, and 1% NEAA at various passages. Cells were successfully expanded until passage 5 and assumed a spindle-shaped fibroblast-like morphology. As the passage increased, polygonal and flatten shaped cells were predominating (a). Rat BM-MSCs were plated in 6-well plate at 150,000 cells/well, and media were changed every 2 days for 6 days. Cells depicted an initial lag phase for 1 day, followed by exponential log phase for 4 days, and then a plateau phase was observed (b). Doubling time was determined by Patterson Formula ∗ and expressed as mean doubling time (c). The result represented the mean ± SD. Microscopic magnification: 200x.

The growth pattern of rBM-MSCs was tracked by the morphological assessment, growth kinetics curve, and doubling time at various passages. The morphology of adherent cells appeared to be relatively smaller and defined at passage 1 till passage 3 (Figure 3(a)). It was noted that cells were assuming a uniform spindle shape till passage 3; however, the morphology has gradually changed from passage 4 onward into a more flatten, larger, and polygonal phenotypes. Although the growth curve of an individual cell passage from passages 1–5 had depicted an accepted sigmoid shape or exponential growth pattern, the duration of lag, log, and plateau phases is varied among numbers of passages (Figure 3(b)). Passages 1–3 cells reflected an initial lag phase for 1 day, followed by exponential log phase for 4 days and then a plateau phase. It was observed that passage 1 showed an abrupt log phase while other passages (P2-3) showed a gradual increase of log phase. However, the proliferative stage of log phase is not being spotted in cells from passages 4-5 which is an indication of growth arrest. The growth arrest of passages 4-5 rBM-MSCs was further confirmed with deduced doubling time. The doubling time of passages 1–3 rBM-MSCs is within 20–30 hours whereas passage 5 shows an extreme of 130 hours as shown in Figure 3(c).

### 3.4. Rat Bone Morrow MSCs Undergo Cellular Senescence and Cell Cycle Arrest

**Figure 4:**
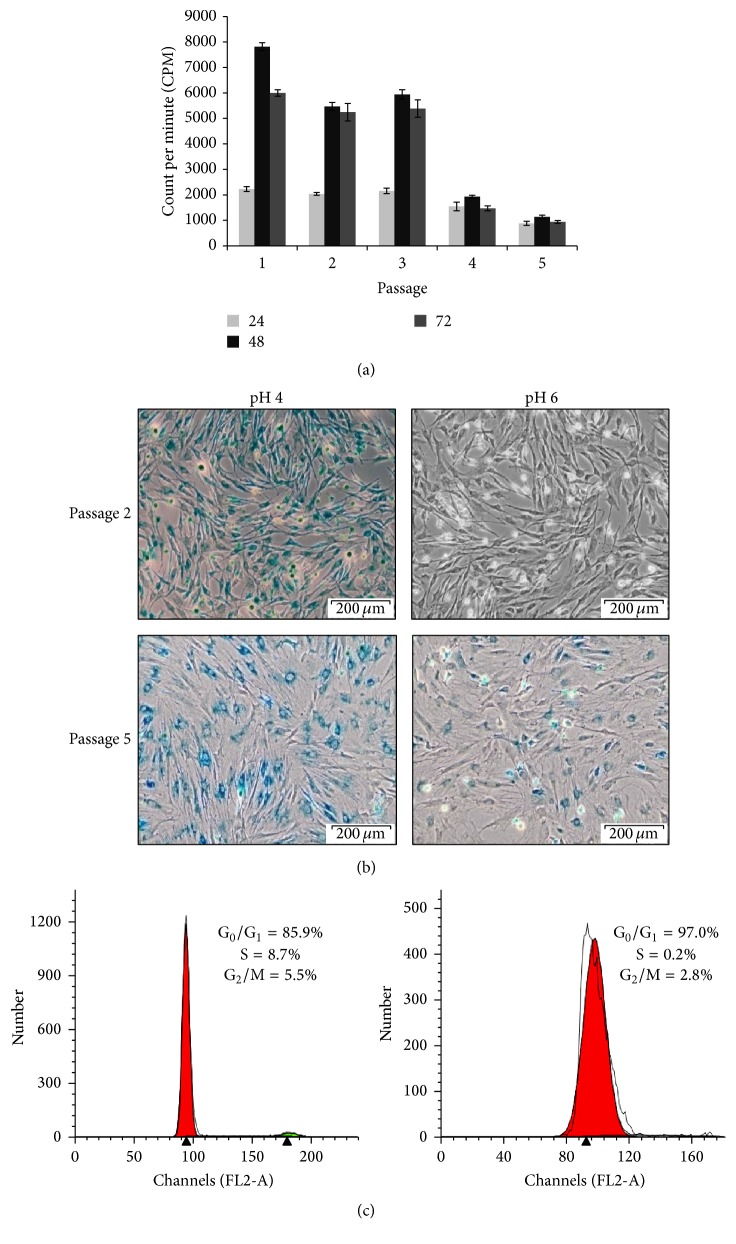
Cellular senescence and cell cycle arrest of culture expanded rBM-MSCs. Cells from passages 1–5 were seeded at 5000 cells/well cultured in 96-well plate for 24 h, 48 h, and 72 h. Cultures were pulsed with 10 *μ*L ^3^H-TdR at 24 hours prior to measurement. The rate of proliferation decreased as the passage increased (a). Cells at passage 2 and passage 5 were fixed with 70% ethanol for 10 minutes and stained with X-gal in the respective buffer overnight. Cells at passages 2 and 5 were stained positive with lysosomal *β*-gal (pH 4) staining showing that cells were expressing lysosomal *β*-gal in early passage as well as in late passage. Cells at passage 5 were stained positive for SA-*β*-gal (pH 6) staining as evidenced by blue precipitation but not at passage 2. Cells at passage 2 and passage 5 were cultured in 25 cm^2^ flasks. Upon reaching 80%–90% confluency, cells were harvested and subjected to PI staining. Cells were analysed using flow cytometer. Results were representative of three independent experiments. Microscopic magnification: 200x.

Since rBM-MSCs from passages 1–5 exhibited various growth kinetic patterns with compromised log phases at late passages, another mean of measurement was opted to verify this phenomenon. The proliferation rate of rBM-MSCs was further rectified using tritiated thymidine (3H-Tdr) incorporation assay. The highest proliferation was observed at 48 hours in all passages. However, the active proliferation was only noted at passages 1–3 while cell expansion was halted at passages 4-5 (Figure 4(a)). The complete halt in cell proliferation at passages 4-5 was further deciphered by the occurrence of senescence. When rBM-MSCs from passage 2 and passage 5 were stained with senescence marker, SA *β*-gal, only passage 5 rBM-MSCs were significantly positive for SA *β*-gal as evidence by blue precipitation within the cells while actively proliferating rBM-MSCs in passage 2 showed negative staining for *β*-gal (pH 6) (Figure 4(b)). To study the cell cycle pattern of rBM-MSC, DNA content of rBM-MSC at passage 2 and passage 5 was measured using PI staining. Result showed that almost all cells in passage 5 were arrested at G_0_/G_1_ phase (G_0_/G_1_ = 97.03%, S = 0.19%, and G_2_/M = 2.78%) as compared to passage 2 (G_0_/G_1_ = 85.85%, S = 8.65%, and G_2_/M = 5.5%) as shown in Figure 4(c).

### 3.5. Senescence of rBM-MSCs Is Not Associated with Apoptosis Induction

Apoptosis assay was performed to assess the viability of rBM-MSCs at various passages (P1-P0) whether the documented cellular senescence is caused by the induction of apoptosis. The apoptosis results revealed that as the passage was increased the percentage of early apoptosis decreased while the percentage of viability is increased as shown in Table 4.

## 4. Discussion

Various studies have been conducted using animal models in attempt to evaluate the potential use of MSCs in clinical applications. As preclinical study is vital for clinical trials, this requires an establishment of animal-based MSCs culture system through a feasible isolation and expansion procedure at in vitro setting [15, 46, 47]. Several methods have been employed to isolate MSCs, mainly based on its plastic adherence property whereby whole bone marrow (BM) is aspirated and directly plated into culture dish. This is the standard method of isolation that is mostly used in previous studies due to the cost effectiveness, straight forward, and less laborious nature of the procedures [48]. Besides plastic adherence, methods of refining MSC population such as density gradient separation technique [42, 49, 50] and enrichment of MSCs culture using magnetic cell sorting [51] were also tested widely. Previous studies have suggested that MSCs isolated from whole BM exhibited the superior cell growth in terms of absolute cell numbers, negligible hematopoietic contamination, higher CFU-f number, and a longer telomere length as compared to Percoll and Ficoll density separation techniques [50]. Hence, the present study has utilized a direct plating method of culturing whole BM from femur and tibia of Sprague Dawley rats.

The initial primary culture (P0) of BM was mixed with other cell populations which did not allow a clear discrimination between MSCs and other adherent cells. However, as the number of passages was increasing, the adherent cell culture became more morphologically homogenous. Along with MSCs, BM cultures at passage 0 were also found to be comprised of other primary BM cells such as macrophages and endothelial cells that promote heterogeneity of primary culture. Since the present study has utilized a whole BM, the contamination of other BM residing cells, namely, hematopoietic cells, fat cells, endothelial cells, and fibroblasts that may retain up to fewer passages of BM culture, could contribute to formation of mixed populations [52]. Nonetheless, mix populations of early bone marrow cells culture are crucial as the growth factors and multicellular interaction with other cells are needed for the initial colony forming and expansion of MSCs [53].

To date, there is no standard method for culturing rBM-MSCs. It is difficult to compare methods of culturing MSCs as there are high inconsistency among different laboratories such as choice of media, the type of serum, plating density, the addition of supplements, and level of confluency which play a crucial role in MSCs culture as it can affect the expansion, differentiation, and immunogenic properties of MSCs [50]. We have employed colony forming unit-fibroblast (CFU-f) assay to optimize the culture condition for rBM-MSC as this assay allows the identification of adherent cells with stem cell properties that expand to form colonies [42]. The current findings demonstrated that LDMEM basal medium with 20% FBS served as the optimal condition for rBM-MSCs expansion, yielding the highest CFU-f count. In line with this, Ayatollahi et al. (2012) had reported the excellent tropic effect of LDMEM basal medium to support and enhance the growth of MSCs as compared to the *α*MEM and HDMEM. Among all tested concentrations of FBS, 15% of supplemented FBS had shown a higher cell yield as compared to the lower concentrations, namely, 5% and 10% of FBS [54]. Since expanding rBM-MSCs from the primary culture are considered an arduous process and thus a higher concentration of FBS, 20% was opted and compared to the conventional 10% of FBS. As expected, 20% of FBS level has induced a higher number of colonies in all media as compared to 10% FBS (Figure 1). However, it is still unable to decipher whether the increased concentration of FBS has potentially slowed down the cellular senescence, although 20% FBS supplemented rBM-MSCs were expanded till passage 4/5 but the conventional FBS concentration fails to propagate cell number at very early passages.

High glucose DMEM and DMEM/f12 are among commonly used media for culturing adherent cells especially MSCs while RPMI are routinely used for culturing lymphocytes [46–60]. In this study, we have also consumed RPMI basal medium to culture rBM-MSC. In fact, rBM-MSCs were able to grow in RPMI although the CFU-f count was lower than LDMEM. Overall, culture medium that contains HDMEM has contributed the lowest number of CFU-f as compared to LDMEM, DMEM/f12, and RPMI. Consumption of the basal medium that contains a high glucose level may jeopardise the beneficiary effects of culturing stem cells over a low glucose culture medium. It has been shown that culturing rat MSCs in high glucose medium is associated with cellular senescence while low glucose medium is enhancing proliferation and CFU counts while reducing the apoptosis [61]. Glucose is crucial source of cellular energy; however, the elevated concentration of glucose can raise the production of reactive oxygen species and thus leads to cell damage and cellular senescence [62]. Due to balanced oxidative stress in low glucose medium, stem cells might be stress-free and physiologically unchallenged since stem cells are very sensitive to the changes in the microenvironment.

Although the colonies formed in LDMEM culture medium are highest, further cultivation in the same medium with 20% FBS did not support the expansion of rBM-MSCs. This phenomenon is also observed in other basal media and thus prompted us to use additional growth factors. Basic FGF was used to enhance the growth of rBM-MSCs in our study as it is the most common growth factor that is known to induce proliferation of MSCs [63–66]. To further improve rBM-MSC growth, 1% ITS and 1% NEAA were also added in culture along with 20 ng/mL bFGF. The addition of bFGF and supplements significantly altered the cell size and appearance of rBM-MSC as compared to rBM-MSC cultured without any supplements (data not shown). It is well established that MSCs at in vitro culture contain two distinct types of morphologies: (1) small spindle-shaped fibroblast-like cells which proliferate rapidly and (2) large flattened cells with slower proliferation rate [16, 46, 50, 57]. Similar to what has been reported, rBM-MSCs cultured in optimized medium and supplements appeared as spindle-shaped and fibroblast-like but when the number of passages increased, a gradual domination of large and flattened cells was noted. Similar observations were also reported by previous studies whereby the number of large and flat cells increased over the time in cultures [49, 57]. This transition could be due to losing of cell integrity and also loss of autocrine and paracrine effects, resulting in inadequate formation of microenvironment that is necessary for MSCs proliferation. Furthermore, it could also be due to an autocommitment of MSCs towards mature mesodermal lineages. Based on RT-PCR data, it has been shown that MSCs at resting condition without inductive media are slightly expressed RNAs that lead to adipogenic and osteogenic differentiation. This could be accountable for the slow growth rate of MSCs during the expansion period where culture-driven cellular commitment towards mature cells reduces the self-renewal and propagation of stem cell population.

Mesenchymal stem cells characterization mainly relies on the assessment of (1) surface antigen markers expression and (2) the ability of MSC to differentiate into mesodermal lineages (osteocytes, adipocytes, and chondrocytes). To date, there are no specific markers for MSCs; thus a combination of commonly accepted positive and negative markers was opted for the MSC's immunophenotyping. We have shown that rBM-MSCs were positive for CD29, CD90.1, CD44H, and CD71 and negative for hematopoietic markers CD45 and CD11b/c [15, 42, 48, 54, 65]. Upon induction, rBM-MSCs were able to differentiate into adipocytes and osteocytes, and this finding was further confirmed by expression of osteocytes (osteopontin and osteonectin) and adipocytes (PPAR*γ* and CEBP/A) genes. Interestingly, rBM-MSCs without the induction medium also expressed a low level of these genes. Others have also reported similar findings where the positive expression of osteonectin and osteopontin and expression of adipogenic gene (PPAR*γ*) were detected in MSCs in the absence of induction medium [16, 67]. The expression of adipogenic and osteogenic genes in undifferentiated rBM-MSCs might be the reason why the cells easily reach senescence and can readily differentiate into mesenchymal stem cells' own mesodermal lineages.

To determine the proliferative capacity of rBM-MSCs, tritiated thymidine (3H-TdR) and growth kinetic assays were performed. Rat BM-MSCs were spotted to proliferate rapidly at passage 1 until passage 3 while reduction in proliferation was evidenced at passage 3 onwards and almost ceased at passage 5. These results were further confirmed with 3H-TdR assay and deduced doubling time. Cells at passages 1–3 had maintained a stable doubling time (30 hrs); however, the doubling time was started to upraise at passage 4 (50 hrs) and reached maximum at passage 5 (130 hrs). However, the cells were unable to be further expanded beyond passage 5 due to the complete halt in cell growth despite the use of optimized culture medium. This observation is not a new phenomenon as Liu et al. (2003) also reported a similar finding that rBM-MSCs from Wister rats were only being cultured till 4 successful passages [49].

The ceased proliferation of passage 5 rBM-MSCs has triggered the need for further exploration of the possibility of apoptosis or senescence. The apoptosis result was not in agreement with the spotted growth arrest of passage 4-5 cells. Surprisingly, the viability of cells was improved with the number of passages. However, a substantial fraction of cells at all passages was undergoing early apoptosis process but not late apoptosis as shown in Table 4. The expression of higher early apoptosis in P1–3 cells was accompanied by neither progression towards late apoptosis nor cell death. However, it could be possible that those cells in early apoptosis are able to recover as the translocation of PS was reversible in apoptosis induced by tumour suppressor protein p53 provided the apoptosis stimuli were removed [68].

Our data also revealed that rBM-MSCs almost cease to proliferate at passage 5 and morphology of cells was large and flattened which are characteristics of cellular senescence. *β*-gal is found in lysosomes of all types of cells at all stages including proliferating, quiescent, and senescence cells and can be detected by X-gal staining at pH 4 [44]. Meanwhile, SA *β*-gal staining is detectable at pH 6.0 and expressed by cells that undergo senescence but not in actively proliferating cells [44, 69]. Result of X-gal staining on passage 2 and passage 5 rBM-MSC showed a positive staining for senescence in passage 5 rBM-MSCs but not in passage 2. Lysosomal *β*-gal assay was also performed on rBM-MSC in passage 2 and passage 5 and our finding showed positive staining for lysosomal *β*-gal. However, since MSCs almost ceased in proliferation as indicated in Figure 3, we were unable to further passage the MSCs and explore the senescence in later passages.

The hallmark of senescence is the inability of cells to advance into cell cycle. Senescence cells show growth arrest at G_1_ phase of cell cycle, despite sufficient growth condition, cells still fail to initiate DNA replication [70]. Cells at passage 5 were mainly arrested at G_0_/G_1_ phase of cell cycle. On the other hand, rBM-MSCs at passage 2 stained which is negative for SA *β*-gal showing a progression in cell cycle with higher percentage of S and G_2_/M phase as compared to passage 5. The reduced proliferation, changes in morphology from spindle-shape fibroblast-like cells into large flattened cells, positive staining for SA *β*-gal, and growth arrest of rBM-MSC passage 5 at G_0_/G_1_ phase of cell cycle could be an evidence of cells undergoing senescence.

Study on human fibroblasts demonstrated that, under an inadequate culture condition (0.25% FBS), the cells underwent a premature growth arrest. When fibroblasts were initially expanded in 10% FBS and later grown in 0.25% FBS, they showed a substantial arrest in cellular growth. However, the cell growth recovered and cell cycle machinery was activated when the culture was reconstituted with 10% FBS. In fact, transfection of HTERT (human telomerase reverse transcriptase) as well did not abolish the growth retardation in the presence of 0.25% of FBS [71]. Regardless of the elongation of telomerase length, serum deprivation elevated p16^INK4a^, a tumour suppressor protein that inhibits cyclin-dependent kinases (CDKs) and leads to a permanent growth arrest [72]. Similarly, human MSCs that underwent a premature growth arrest during the in vitro culture as well showed a significant change in p16^INK4a^ level [73]. It was elegantly shown that the suppression of p16^INK4a^ in human MSCs by wild-type p53 inducible phosphatase-1 (Wip-1), a stress modulator, extended the life-span of human MSCs while maintaining its multipotent differentiation potential [73].

In conclusion, the present study has successfully generated and characterized the rBM-MSCs from SD rats with optimized culture conditions based on the choice of basal medium, concentration of FBS, and growth factor supplements. The basal media LDMEM and 20% FBS provide the optimal culture condition for expanding rBM-MSC and the addition of bFGF, ITS, and NEAA significantly enhanced the morphology and proliferation capacity of rBM-MSCs at very early passages. Under the in vitro culture condition, even with optimized culture conditions, rBM-MSCs were undergoing cellular senescence which may relate to the gradual autocommitment of rBM-MSC into mature cells. Thus, further research is necessary to understand the internal and external cues that trigger the process of senescence in culture expanded rBM-MSCs.

## Figures and Tables

**Table 1:** Basal media and FBS concentration used for CFU-f assay.

Basal media	FBS concentration (%)
Roswell Park Memorial Institute (RPMI) with GLUTAMAX-I	10
RPMI with GLUTAMAX-I	20
Dulbecco's Modified Eagle's Medium with nutrient mixture F12 (HAM) [1 : 1] DMEM/F12) with GLUTAMAX-I	10
DMEM/F12 with GLUTAMAX-I	20
Low glucose Dulbecco's Modified Eagle's Medium (LDMEM) with GLUTAMAX-I	10
LDMEM with GLUTAMAX-I	20
High glucose Dulbecco's Modified Eagle Medium (HDMEM) with GLUTAMAX-I	10
HDMEM with GLUTAMAX-I	20

**Table 2:** Primer sequence for adipocyte and osteocytes.

Primer	5′-3′	Sequence	Amplicon size	Annealing temperature (°C)
GAPDH	Forward Reverse	TGAACGGGAAGCTCACTGG TCCACCACCCTGTTGCTGTA	360	48.1
Osteopontin	Forward Reverse	CCGATGAATCTGATGAGTCCTT TCCAGCTGACTTGACTCATGG	303	57.8
Osteonectin	Forward Reverse	ATGAGGGCCTGGATCTTCTTTCTC AAAGAAGTGGCAGGAAGAGTCGA	372	60.5
PPAR*γ*	Forward Reverse	GCCTTGCTGTGGGGATGTCT CGAAACTGGCACCCTTGAAAAAT	354	47.3
C/EBPA	Forward Reverse	GCAGAAGGTGTTGGAGTTGA AGCGACCCTAAACCATCCTC	214	66.7

**Table 3:** PCR gene amplification conditions for GAPDH, osteopontin, osteonectin, PPAR*γ*, and C/EBPA gene for adipogenesis and osteogenesis differentiation assays.

Process	Temperature (°C)	Time	Cycle
Predenaturation	95	3 min	1
Denaturation	95	30 sec	35
Annealing		1 min	35
GAPDH	48.1		
Osteopontin	57.8		
Osteonectin	60.5		
PPAR*γ*	47.3		
C/EBPA	66.7		
Elongation	72	1 min	35
Final elongation	72	10 min	1
Incubation	10	∞	∞

**Table 4:** Percentage of viability and early apoptosis at various passages of rBM-MSC.

Passage	Viable cells (%)	Early apoptosis (%)
1	30.1 ± 1.0	68.4 ± 0.4
2	42.4 ± 3.0	56.8 ± 3.0
3	49.9 ± 4.0	49.6 ± 4.0
4	67.2 ± 3.0	31.2 ± 2.0
5	69.4 ± 1.0	29.4 ± 0.5
